# Clinico-Biochemical Profile and Outcomes in Young Adults With ST-Segment Elevation Myocardial Infarction at a Tertiary Care Hospital: An Observational Study

**DOI:** 10.7759/cureus.111771

**Published:** 2026-06-29

**Authors:** Jeyanth K, Kavitha B, Surendar R, Rajalakshmi E, Mukilan S, Sreeshitha CM, Madumitha Pandurangan

**Affiliations:** 1 Medicine, Indira Gandhi Medical College and Research Institute, Puducherry, IND; 2 Internal Medicine, Indira Gandhi Medical College and Research Institute, Puducherry, IND; 3 Emergency Medicine, Jawaharlal Institute of Postgraduate Medical Education & Research, Puducherry, IND; 4 General Medicine, Indira Gandhi Medical College and Research Institute, Puducherry, IND; 5 Emergency Medicine, Indira Gandhi Medical College and Research Institute, Puducherry, IND

**Keywords:** myocardial infarction, thrombolytic therapy, time-to-treatment, treatment outcome, young adult

## Abstract

Background

ST-elevation myocardial infarction (STEMI) in young adults is increasingly recognized, with distinct risk factor profiles and potential differences in clinical outcomes compared to older patients. However, data from the Indian context remain limited, particularly regarding thrombolysis response.

Objectives

The aim of this study was to compare the clinical profile, risk factors, laboratory parameters, and outcomes of STEMI between young adults (≤45 years) and older patients (>45 years), with a focus on thrombolysis outcomes.

Methods

This prospective observational study included 60 patients with STEMI admitted to a tertiary care center over six months. Patients were categorized into young (≤45 years, n=16) and older (>45 years, n=44) groups. Clinical presentation, risk factors, laboratory parameters, electrocardiographic findings, thrombolysis characteristics, and outcomes were compared. Statistical analysis was performed using the chi-square test for categorical variables, with p<0.05 considered significant.

Results

Young adults constituted 16 (26.7%) cases and were predominantly male (45, 75.0%). Smoking was significantly more prevalent among younger patients (seven, 43.8% vs seven, 15.9%, p=0.024), while diabetes mellitus was more common in older patients (24, 54.5% vs four, 25.0%, p=0.042). Clinical presentation and ECG patterns were comparable between the groups. Younger patients had significantly higher white blood cell counts (11993.75 ± 3630.70 vs 9479.55 ± 2791.23 /µL, p=0.006) and platelet counts (312250 ± 75964.47 vs 261045.45 ± 60385.18 /µL, p=0.009). Despite similar door-to-needle times, successful thrombolysis was significantly lower among younger patients (seven, 43.8% vs 33, 75.0%, p=0.023). Overall clinical outcomes were comparable between the groups (p=0.227).

Conclusion

Young STEMI patients exhibit a distinct risk profile dominated by modifiable factors and demonstrate reduced thrombolysis success despite timely reperfusion, suggesting a heightened prothrombotic and inflammatory state. These findings underscore the importance of targeted prevention and support consideration of early invasive strategies in younger patients, warranting further large-scale studies.

## Introduction

Cardiovascular diseases (CVDs) remain the leading cause of mortality worldwide, accounting for over 20 million deaths annually, with more than 80% occurring in developing countries [1]. Among these, coronary artery disease (CAD) and stroke contribute significantly to the global burden. Acute coronary syndrome (ACS), as classified by contemporary guidelines, encompasses unstable angina, non-ST elevation myocardial infarction (NSTEMI), and ST-elevation myocardial infarction (STEMI), with STEMI representing the most severe form due to complete or near-complete coronary artery occlusion leading to myocardial necrosis [2].

STEMI is primarily diagnosed based on characteristic electrocardiographic changes, particularly ST-segment elevation, in conjunction with clinical symptoms and elevation of cardiac biomarkers such as troponins [2]. It continues to be a major contributor to morbidity and mortality, with an estimated global prevalence of over three million cases annually [3]. Recent data indicate a rising incidence of STEMI in low- and middle-income countries, including India and China, where it accounts for a substantial proportion of acute myocardial infarction hospitalizations [3].

In recent years, there has been a noticeable increase in the incidence of myocardial infarction (MI) among younger individuals. This trend has been attributed to lifestyle-related factors such as smoking, obesity, unhealthy dietary habits, physical inactivity, and psychosocial stress [4,5]. Traditionally, MI in individuals aged ≤45 years has been categorized as “young MI,” although varying definitions have been used across studies [6]. Young STEMI patients often differ from older patients in terms of risk factor profile, pathophysiology, and clinical outcomes, with a relatively lower prevalence of traditional co-morbidities but a higher burden of modifiable risk factors.

Early diagnosis and timely reperfusion remain the cornerstone of STEMI management, with primary percutaneous coronary intervention (PCI) being the preferred strategy. In situations where timely PCI is not feasible, fibrinolytic therapy is recommended to restore coronary perfusion [7]. Prompt intervention significantly reduces infarct size, preserves myocardial function, and improves survival outcomes.

Although primary PCI remains the preferred reperfusion strategy, fibrinolytic therapy continues to play a major role in STEMI management across many parts of India because of delays in access to PCI-capable centers and resource limitations. Timely and successful thrombolysis is critical for myocardial salvage; however, failed reperfusion remains an important clinical challenge associated with recurrent ischemia, larger infarct size, heart failure, and increased mortality. Recent Indian data have highlighted ongoing variations in reperfusion success and clinical outcomes among STEMI patients receiving thrombolytic therapy. While young STEMI patients are increasingly recognized as a distinct subgroup with unique risk factor profiles and clinical characteristics, evidence regarding thrombolysis response and short-term outcomes in this population remains limited. Furthermore, direct comparisons of reperfusion success between young and older STEMI patients in the Indian setting are scarce [8,9]. 

Given these gaps in the literature, particularly regarding age-related differences in thrombolysis response and short-term outcomes in the Indian setting, this study was undertaken to compare the clinical and biochemical profiles of young and older STEMI patients and to evaluate thrombolysis response and short-term clinical outcomes following fibrinolytic therapy.

## Materials and methods

Study design and setting

This was a prospective observational study conducted at Indira Gandhi Medical College and Research Institute (IGMCRI), a tertiary care teaching hospital in Puducherry, India. The study was carried out in the emergency medical services (EMS) and intensive care unit (ICU) of the institution over a period of six months, from April 2025 to September 2025.

Study population

All patients aged ≥18 years presenting with acute STEMI during the study period were screened for eligibility. A total of 77 patients were initially assessed, of whom 60 patients met the inclusion criteria and were enrolled in the study. The study population was stratified into two groups based on age: young adults (≤45 years) and older patients (>45 years).

Inclusion and exclusion criteria

All eligible patients were screened according to predefined inclusion and exclusion criteria. The criteria used for participant selection are summarized in Table 1.

**Table 1:** Inclusion and exclusion criteria of study participants

Inclusion criteria	Exclusion criteria
Age ≥18 years	Non-ST elevation myocardial infarction (NSTEMI)
Diagnosed with acute ST-elevation myocardial infarction (STEMI)	Pericarditis
ST-segment elevation in two contiguous leads on electrocardiography	Myopericarditis
Elevated cardiac biomarkers consistent with STEMI	Aortic dissection
Provided written informed consent	Congenital heart disease
	Pregnancy
	Age <18 years

Patients were included if they were aged 18 years or older and diagnosed with acute STEMI based on standard criteria. STEMI was defined according to contemporary European Society of Cardiology (ESC) guidelines as new ST-segment elevation at the J point in at least two contiguous leads, with cutoff values of ≥2.5 mm in men aged <40 years, ≥2.0 mm in men aged ≥40 years, or ≥1.5 mm in women in leads V2-V3 and/or ≥1.0 mm in other contiguous leads, in the appropriate clinical setting, with elevation of cardiac biomarkers consistent with myocardial necrosis [10].

Patients with conditions that could mimic STEMI or confound the diagnosis were excluded. These included NSTEMI, pericarditis, myopericarditis, aortic dissection, congenital heart diseases, pregnancy, age below 18 years, and those who did not provide consent to participate in the study.

Data collection

A semi-structured proforma was used to collect demographic details, clinical presentation, risk factors, and past medical history. Data regarding clinical findings, laboratory investigations, electrocardiographic patterns, and treatment details were obtained from patient records. Information regarding prior COVID-19 infection and COVID-19 vaccination status was also collected because both have been reported to influence thrombo-inflammatory pathways and cardiovascular events, potentially affecting the clinical presentation and outcomes of acute myocardial infarction. Body mass index (BMI) was categorized according to the World Health Organization classification as normal weight (<25 kg/m²), overweight (25.0-29.9 kg/m²), and obese (≥30 kg/m²).

All patients were managed according to institutional STEMI treatment protocols based on contemporary guideline recommendations. Patients presenting within the eligible time window and without contraindications to fibrinolysis received thrombolytic therapy using streptokinase. Standard medical therapy included dual antiplatelet therapy (aspirin and clopidogrel), anticoagulation, high-intensity statin therapy, and other guideline-directed medications such as beta-blockers and angiotensin-converting enzyme inhibitors or angiotensin receptor blockers when clinically indicated. Treatment decisions were made by the attending physicians, independent of study participation. Additional investigations performed for study purposes included complete blood count and renal profile, obtained after informed consent. 

Thrombolysis and treatment protocol

Reperfusion therapy was administered as per standard guidelines. Thrombolysis was performed in eligible patients using tenecteplase as the primary thrombolytic agent and was administered as a single weight-adjusted intravenous bolus (30-50 mg according to body weight). Streptokinase, when used, was administered at a dose of 1.5 million units intravenously over 60 minutes. Tenecteplase was the preferred thrombolytic agent because of its ease of administration as a single bolus and established efficacy. Streptokinase was used in selected cases based on physician discretion and institutional considerations such as availability and cost, reflecting routine clinical practice. Door-to-needle (DTN) time was recorded for all patients.

Successful thrombolysis was defined as ≥50% resolution of the maximal baseline ST-segment elevation on repeat electrocardiography performed 90 minutes after administration of fibrinolytic therapy, consistent with guideline-based criteria for successful reperfusion. Clinical improvement and relief of ischemic symptoms were recorded separately and were not included in the primary definition of successful thrombolysis [10].

Outcome measures and follow-up

Patients were followed up for a period of 30 days from the time of admission. Follow-up was conducted through telephonic communication. Clinical outcomes were categorized as recovery without adverse cardiovascular events (ACVE), recovery with ACVE, including stroke, reinfarction, and acute kidney injury, in-hospital death, and death after discharge.

Statistical analysis

Data were entered into Microsoft Excel (Microsoft Corporation, Redmond, WA, USA) and analyzed using IBM SPSS Statistics software, version 27.0 (IBM Corp., Armonk, NY, USA) [11]. Continuous variables were assessed for normality using the Shapiro-Wilk test. Normally distributed continuous variables were expressed as mean ± standard deviation and compared using the independent samples t-test, whereas non-normally distributed variables were compared using the Mann-Whitney U test. Categorical variables were expressed as frequencies and percentages and compared using the chi-square test or Fisher’s exact test, as appropriate. Fisher’s exact test was used when expected cell counts were less than five. A p-value <0.05 was considered statistically significant. 

Ethical considerations

The study protocol was approved by the Institutional Ethics Committee (approval No: 691/IEC-42/IGMC&RI/PP-39/2024). Written informed consent was obtained from all participants prior to inclusion in the study. The study was observational in nature, and data collection did not interfere with routine patient management, treatment decisions, or follow-up. All patients received standard care as determined by the treating physicians in accordance with institutional protocols.

This observational study was reported in accordance with the Strengthening the Reporting of Observational Studies in Epidemiology (STROBE) guidelines.

## Results

**Figure 1:**
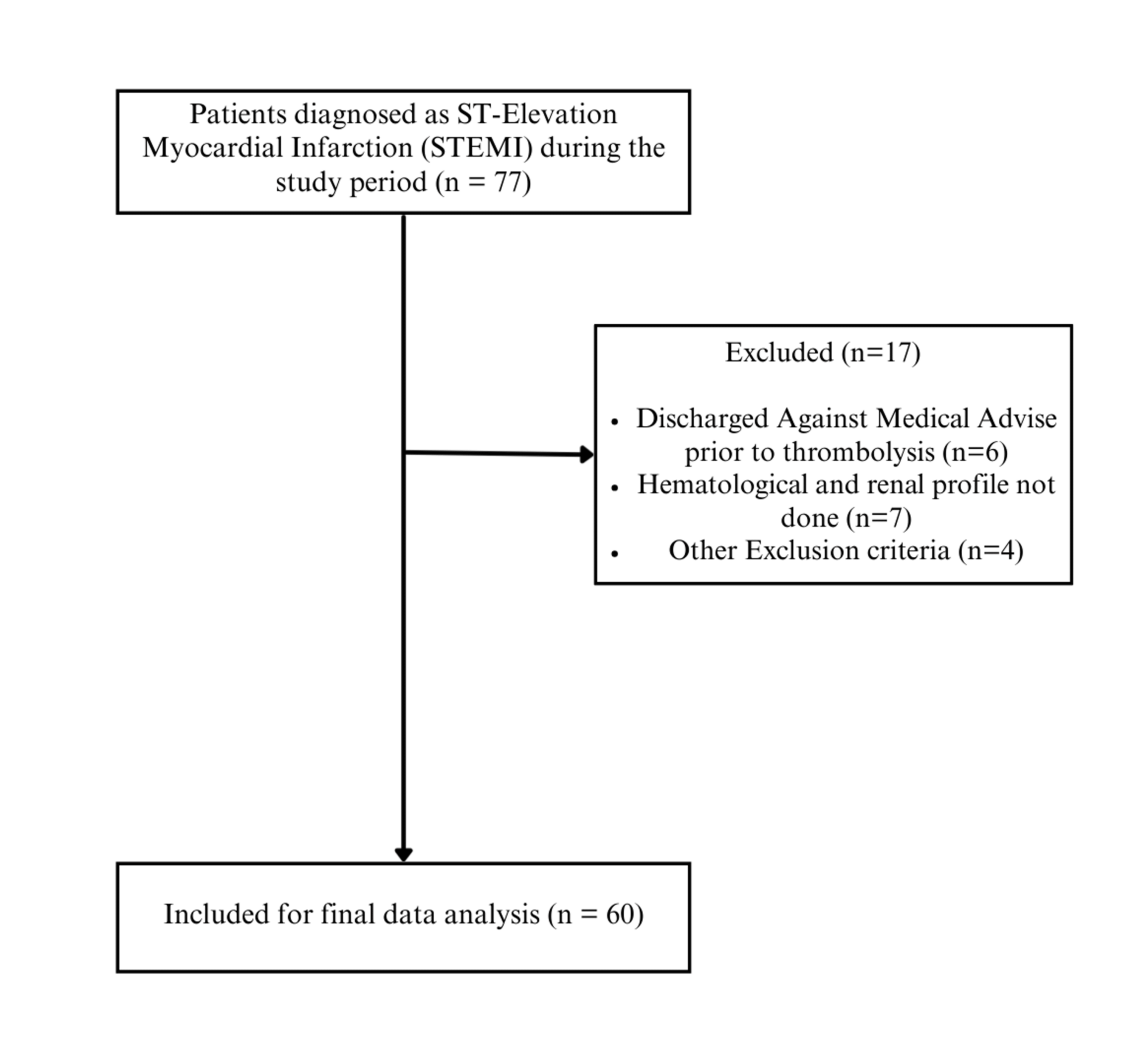
Flow diagram of patient recruitment, enrollment, follow-up, and analysis.

A total of 77 patients were assessed for eligibility during the study period. Seventeen patients were excluded, and 60 patients meeting the eligibility criteria were enrolled in the study. Of these, 16 (26.7%) were classified as young STEMI patients (≤45 years) and 44 (73.3%) as older STEMI patients (>45 years). All enrolled patients completed the 30-day follow-up and were included in the final analysis (Figure 1).

Demographic characteristics of the study population

A total of 60 patients with STEMI were included in the study. Of these, 16 (26.7%) were young adults (≤45 years), while 44 (73.3%) were older patients (>45 years). The overall mean age of the study population was 53.92 ± 13.06 years, with a range of 24 to 79 years. The mean age in the younger group was 38.44 ± 7.15 years, whereas in the older group it was 59.55 ± 9.73 years. Male predominance was observed, accounting for 45 (75%) of cases (Table 2).

**Table 2:** Demographic characteristics of the study population (N=60)

Variable	Category	n (%)
Age group	≤45 years	16 (26.7%)
	>45 years	44 (73.3%)
Gender	Male	45 (75.0%)
	Female	15 (25.0%)

Clinical presentation of STEMI by age group

Chest pain was the most common presenting symptom in both young and older patients, occurring in 15 (93.8%) younger patients and 42 (95.5%) older patients (Table 3). Other symptoms, such as dyspnea, palpitations, syncope, and gastrointestinal complaints, were comparable between the two groups, with no statistically significant differences. Sweating was more frequently observed in younger patients, occurring in 11 (68.8%) compared to 18 (40.9%) older patients, although this did not reach statistical significance (p=0.056).

**Table 3:** Clinical presentation of ST-elevation myocardial infarction (STEMI) patients by age group (N=60)

Symptom	Young (≤45 years) n (%)	Older (>45 years) n (%)	Chi-square (χ²,df)	p-value	Odds ratio (95% CI)
Chest pain	15 (93.8)	42 (95.5)	0.072 (1)	0.789	1.40 (0.12–16.58)
Dyspnea	9 (56.3)	20 (45.5)	0.548 (1)	0.459	0.65 (0.21–2.05)
Palpitations	7 (43.8)	24 (54.5)	0.548 (1)	0.459	1.54 (0.49–4.88)
Syncope	1 (6.3)	8 (18.2)	1.310 (1)	0.252	3.33 (0.38–29.03)
Sweating	11 (68.8)	18 (40.9)	3.642 (1)	0.056	0.32 (0.09–1.06)
Radiating pain (arm/jaw/shoulder)	10 (62.5)	36 (81.8)	2.448 (1)	0.118	2.70 (0.76–9.61)
GI symptoms	6 (37.5)	14 (31.8)	0.170 (1)	0.680	0.78 (0.24–2.57)

Comorbidities among STEMI patients by age group

Diabetes mellitus was significantly more prevalent among older patients compared to younger patients, occurring in 24 (54.5%) versus four (25.0%) patients (p=0.042) (Table 4). Hypertension was also more common in the older group (21, 47.7% vs five, 31.3%), although the difference was not statistically significant (p=0.255). Pre-existing ischemic heart disease was observed only among older patients, but did not show a significant difference between groups.

**Table 4:** Co-morbidities among ST-elevation myocardial infarction (STEMI) patients by age group (N=60) *Statistically significant (p < 0.05)

Comorbidity	Young (≤45 years), n (%)	Older (>45 years), n (%)	Chi-square (χ², df)	p-value	Odds ratio (95% CI)
Hypertension	5 (31.3)	21 (47.7)	1.297 (1)	0.255	2.01 (0.60–6.75)
Diabetes mellitus	4 (25.0)	24 (54.5)	4.115 (1)	0.042*	3.60 (1.00–12.92)
Ischemic heart disease	0 (0.0)	2 (4.5)	0.752 (1)	0.386	1.05 (0.98–1.12)

Risk factors and BMI among STEMI patients by age group

Smoking was significantly more prevalent among younger patients, seen in seven (43.8%) compared to seven (15.9%) older patients (p=0.024) (Table 5). Alcohol use was also higher in the younger group (10, 62.5% vs 17, 38.6%), although this difference was not statistically significant (p=0.100). The majority of patients in both age groups were either overweight or obese, with no significant difference in BMI distribution between the groups (p=0.206).

**Table 5:** Risk factors and body mass index among ST-elevation myocardial infarction (STEMI) patients by age group *Statistically significant (p < 0.05)

Variable	Category	Young (≤45 years, N=16) n (%)	Older (>45 years, N=44) n (%)	Chi-square (χ², df)	p-value	Odds ratio (95% CI)
Smoking	Yes	7 (43.8)	7 (15.9)	5.084 (1)	0.024*	0.24 (0.07–0.87)
Alcohol use	Yes	10 (62.5)	17 (38.6)	2.700 (1)	0.100	0.38 (0.12–1.23)
Body mass index	Normal	3 (18.8)	16 (36.4)	3.158 (2)	0.206	—
	Overweight	9 (56.3)	14 (31.8)			
	Obese	4 (25.0)	14 (31.8)			

COVID-19 infection and vaccination status among STEMI patients by age group

A high proportion of patients in both age groups were vaccinated against COVID-19, with slightly higher coverage among older patients (42, 95.5% vs 14, 87.5%), although this difference was not statistically significant (p=0.275) (Table 6). Similarly, prior COVID-19 infection rates were comparable between the two groups (9, 20.5% vs 3, 18.8%), with no significant difference (p=0.884).

**Table 6:** COVID-19 infection and vaccination status among ST-elevation myocardial infarction (STEMI) patients by age group

Variable	Category	Young (≤45 years, N=16) n (%)	Older (>45 years, N=44) n (%)	Chi-square (χ²)	p-value	Odds ratio (95% CI)
COVID-19 vaccination	Yes	14 (87.5)	42 (95.5)	1.193	0.275	3.00 (0.39–23.33)
COVID-19 infection	Yes	3 (18.8)	9 (20.5)	0.021	0.884	1.11 (0.26–4.77)

Killip classification of STEMI patients by age group

**Figure 2:**
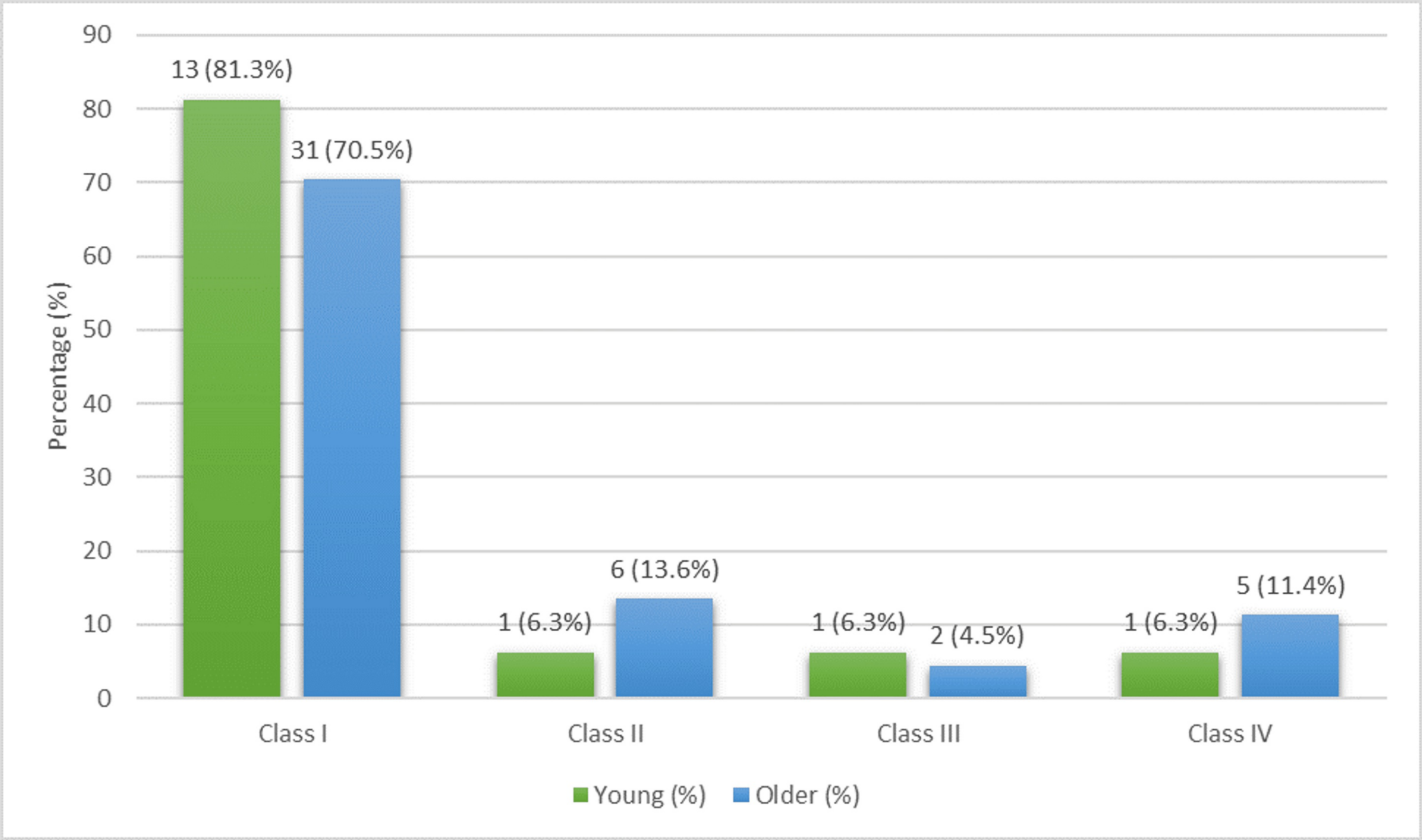
Distribution of Killip classification among ST-elevation myocardial infarction (STEMI) patients by age group

Most patients in both age groups presented with Killip Class I, accounting for 13 (81.3%) younger patients and 31 (70.5%) older patients. Killip Class II was observed in one (6.3%) younger patient and six (13.6%) older patients. Higher Killip classes, including Class III and IV, were relatively more frequent among older patients, with Class IV occurring in one (6.3%) younger patient and five (11.4%) older patients. Overall, there was no statistically significant difference in Killip class distribution between the two groups (p=0.775) (Figure 2).

ECG patterns among STEMI patients by age group

**Figure 3:**
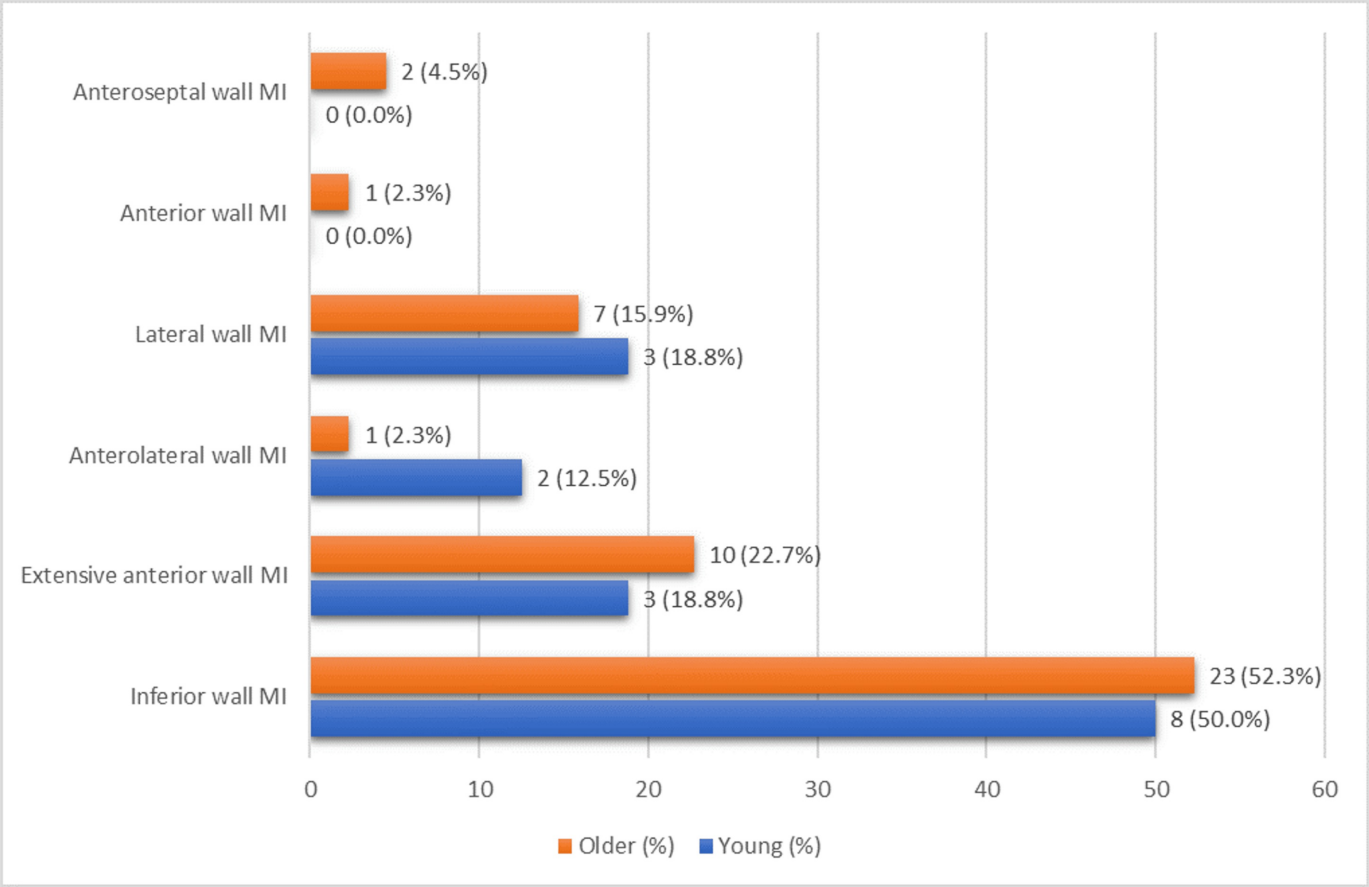
Distribution of ECG patterns among ST-elevation myocardial infarction (STEMI) patients by age group MI: myocardial infarction

Inferior wall MI was the most common ECG presentation in both young and older patients, occurring in eight (50.0%) younger patients and 23 (52.3%) of older patients. Extensive anterior wall MI was observed in three (18.8%) younger patients and 10 (22.7%) older patients, while anterolateral and lateral wall infarctions were less frequent in both groups. Anterior and anteroseptal wall MI were observed only among older patients. Overall, there was no statistically significant difference in the distribution of ECG patterns between the two groups (p=0.593) (Figure 3).

Clinical and laboratory parameters among STEMI patients by age group

Clinical parameters, including pulse rate and blood pressure, were broadly comparable between the two groups. Younger patients had a higher mean pulse rate (88.75 ± 14.27 beats per minute (bpm); range 74-133) compared to older patients (79.23 ± 17.64 bpm; range 23-120), although this difference approached but did not reach statistical significance (p=0.057). Similarly, systolic and diastolic blood pressures were higher in younger patients but did not differ significantly between groups.

Among hematological parameters, younger patients demonstrated significantly higher white blood cell counts (11993.75 ± 3630.70 /µL; range 7500-20900) compared to older patients (9479.55 ± 2791.23 /µL; range 5400-19800) (p=0.006). Platelet counts were also significantly elevated in the younger group (312250 ± 75964.47 /µL; range 160000-386000) compared to the older group (261045.45 ± 60385.18 /µL; range 161000-380000) (p=0.009). Other hematological indices, including hemoglobin and differential leukocyte counts, were comparable between the groups.

Renal and biochemical parameters, including urea, creatinine, and serum electrolytes, showed no statistically significant differences between younger and older patients (Table 7).

**Table 7:** Clinical and laboratory parameters among ST-elevation myocardial infarction (STEMI) patients by age group *Statistically significant (p < 0.05)

Parameter	Young (≤45 years, N=16) Mean ± SD	Older (>45 years, N=44) Mean ± SD	p-value
Clinical parameters			
Pulse rate (beats per minute)	88.75 ± 14.27	79.23 ± 17.64	0.057
Systolic blood pressure (BP, mmHg)	141.25 ± 33.64	131.55 ± 22.63	0.205
Diastolic BP (mmHg)	97.50 ± 32.76	83.77 ± 13.77	0.123
Hematological parameters			
Hemoglobin (g/dL)	13.53 ± 2.92	13.63 ± 2.17	0.884
RBC (×10⁶/µL)	4.76 ± 0.59	4.74 ± 0.51	0.866
WBC (/µL)	11993.75 ± 3630.70	9479.55 ± 2791.23	0.006*
Platelets (/µL)	312250 ± 75964.47	261045.45 ± 60385.18	0.009*
Neutrophils (%)	72.63 ± 9.49	69.61 ± 10.40	0.315
Eosinophils (%)	1.31 ± 1.01	1.20 ± 0.79	0.668
Lymphocytes (%)	23.56 ± 9.19	26.36 ± 9.80	0.324
Monocytes (%)	2.50 ± 0.89	2.82 ± 1.53	0.437
Renal and biochemical parameters			
Urea (mg/dL)	26.81 ± 15.03	26.61 ± 7.44	0.946
Creatinine (mg/dL)	1.03 ± 0.98	0.85 ± 0.26	0.490
Sodium (mmol/L)	137.69 ± 4.67	139.64 ± 3.25	0.074
Potassium (mmol/L)	4.35 ± 0.69	4.24 ± 0.54	0.509
Chloride (mmol/L)	101.31 ± 5.07	101.30 ± 4.00	0.989

DTN time and thrombolysis outcomes among STEMI patients by age group

All patients in the younger age group were thrombolysed within one hour of arrival, with a mean DTN time of 32.38 ± 2.96 minutes, which was comparable to that of older patients (32.86 ± 3.76 minutes). A small proportion of patients experienced adverse effects following thrombolysis, occurring in one (6.3%) younger patient and three (6.8%) older patients, with no significant difference between the groups (p=0.938) (Table 8).

**Table 8:** Thrombolysis characteristics and outcomes among ST-elevation myocardial infarction (STEMI) patients by age group *Statistically significant (p < 0.05)

Variable	Category	Young (≤45 years, N=16) n (%)	Older (>45 years, N=44) n (%)	χ²(df)	p-value
Adverse effects	Yes	1 (6.3)	3 (6.8)	0.006 (1)	0.938
Successful thrombolysis*	Yes	7 (43.8)	33 (75.0)	5.156 (1)	0.023*

Successful thrombolysis was achieved in seven (43.8%) younger patients compared to 33 (75.0%) older patients, demonstrating a statistically significant difference (p=0.023). Conversely, failed thrombolysis was more frequent among younger patients (9, 56.3% vs 11, 25.0%).

Clinical outcomes among STEMI patients by age group

With respect to clinical outcomes, the majority of patients in both age groups recovered without adverse cardiovascular events, accounting for 13 (81.3%) younger patients and 36 (81.8%) older patients (Table 9). Recovery with adverse events was observed in two (12.5%) younger patients and four (9.1%) older patients. In-hospital mortality occurred in one (6.3%) younger patient, while post-discharge mortality was observed only among older patients (four, 9.1%). During the 30-day follow-up period, no statistically significant difference in clinical outcomes was observed between the younger and older groups (p=0.227); however, the relatively small sample size limits the ability to detect clinically meaningful differences in adverse events.

**Table 9:** Clinical outcomes among STEMI patients by age group

Outcome	Young (≤45 years, N=16) n (%)	Older (>45 years, N=44) n (%)	Chi-square (χ², df)	p-value
Recovered without adverse cardiovascular events (ACVE)	13 (81.3)	36 (81.8)	4.341 (3)	0.227
Recovered with ACVE	2 (12.5)	4 (9.1)		
In-hospital death	1 (6.3)	0 (0.0)		
Death after discharge	0 (0.0)	4 (9.1)		

## Discussion

Demographic profile

In this study, the clinical profile, risk factors, and outcomes of STEMI were compared between young adults (≤45 years) and older patients (>45 years). The mean ages of patients in the young and older groups were 38.4 ± 7.1 years and 59.55 ± 9.7 years, respectively. The overall mean age of the study population was 53.92 ± 13.06 years, which is comparable to reports from South Asian populations where MI occurs at a younger age compared to Western cohorts [12].

Young adults constituted 26.67% of the study population, which is higher than some international reports but comparable to Indian registry data [13,14]. The study population showed a clear male predominance (75%), consistent with previous studies demonstrating a higher incidence of STEMI among men, particularly in younger age groups [12-16].

Clinical presentation

Chest pain was the most common presenting symptom in both age groups, consistent with previous literature [17,18]. Sweating was more frequently observed among younger patients, while other symptoms such as breathlessness, palpitations, syncope, and gastrointestinal complaints were comparable between groups. Prior studies have similarly reported chest pain and autonomic symptoms as the predominant presentation in young STEMI patients [19]. The absence of significant differences suggests that clinical presentation alone may not reliably distinguish between age groups.

Risk factors and comorbidities

Hypertension was more common among older patients, while diabetes mellitus was significantly more prevalent in the older age group, reflecting the known age-related increase in metabolic risk factors [17,20,21]. However, some studies have reported a high prevalence of diabetes even among younger Indian patients, suggesting regional variability in risk factor distribution [22].

Smoking emerged as a major risk factor among younger patients, with a significantly higher prevalence compared to older patients. This finding is consistent with large international and regional studies that have identified smoking as the most important modifiable risk factor in young MI [12-16,22]. The high prevalence of overweight and obesity in both groups further reflects the contribution of lifestyle-related factors in the pathogenesis of STEMI.

COVID-19 vaccination status and prior COVID-19 infection were also evaluated as exploratory variables because of the recognized association between COVID-19-related inflammation, endothelial dysfunction, and prothrombotic states. In the present study, no significant differences were observed between young and older STEMI patients with respect to COVID-19 vaccination status or prior infection. However, detailed information regarding vaccine type, number of doses, timing of infection, and disease severity was not collected. Therefore, these findings should be interpreted cautiously, and further studies are required to better understand the potential influence of COVID-19-related factors on STEMI presentation and reperfusion outcomes [23].

ECG patterns and disease characteristics

Inferior wall MI was the most common ECG presentation in both age groups, consistent with prior studies [23,24]. However, some studies have reported anterior wall infarction as more common, indicating variability based on population characteristics [25]. Previous literature also suggests that young STEMI patients more commonly have single-vessel disease, often involving the left anterior descending artery, with plaque erosion and a prothrombotic milieu playing a significant role in pathogenesis [23,26-28].

Laboratory findings

Younger patients demonstrated significantly higher white blood cell and platelet counts compared to older patients. Leukocytosis is a recognized marker of inflammation in acute myocardial infarction and has been associated with adverse outcomes [29]. Elevated platelet counts and activity reflect increased platelet aggregation and thrombus formation, which may contribute to coronary occlusion [30]. These findings may reflect differences in inflammatory or thrombotic activity between younger and older patients; however, specific inflammatory biomarkers, platelet function markers, and angiographic thrombus burden were not assessed in the present study. Further studies incorporating inflammatory biomarkers such as high-sensitivity C-reactive protein and procalcitonin would be required to better characterize this association.

Thrombolysis outcomes

A key finding of this study was the significantly lower success rate of thrombolysis among younger patients compared to older patients. Despite comparable DTN times, thrombolysis success was reduced in younger individuals, indicating that factors beyond treatment delay may be involved.

The pathophysiology of STEMI in younger patients is often characterized by plaque erosion, increased platelet reactivity, and a prothrombotic state, which may result in thrombi that are less responsive to fibrinolytic therapy [26-28,30]. The significantly higher leukocyte and platelet counts observed in younger patients in this study further support this hypothesis. Additionally, smoking, more prevalent in the younger group, is known to impair endothelial function and fibrinolysis [12,22]

In contrast, some studies have reported better thrombolysis outcomes in younger patients, suggesting that regional variations, differences in thrombus composition, lipid profiles, and treatment strategies may influence reperfusion response [30]. In the present study, younger patients demonstrated a distinct cardiovascular risk profile, which may have contributed to the observed variation in thrombolysis success; however, the underlying mechanisms remain uncertain and warrant further investigation.

Clinical outcomes

Despite differences in thrombolysis success, overall clinical outcomes were comparable between the two groups, with the majority of patients recovering without adverse cardiovascular events. Mortality rates were low and consistent with previous reports among young STEMI patients [9,15,16,27]. Established predictors of mortality include reduced left ventricular ejection fraction, diabetes, smoking, and delayed or absent revascularization [9].

Implications

The findings of this study highlight the distinct risk factor profile and thrombolysis response in young STEMI patients. The observed burden of modifiable cardiovascular risk factors, particularly smoking, among younger STEMI patients highlights the importance of preventive cardiovascular health initiatives and risk factor modification in this population. Additionally, the reduced success of thrombolysis in younger patients, despite timely administration, suggests that early invasive strategies such as primary PCI or pharmaco-invasive approaches may be particularly beneficial in this subgroup, where feasible.

This study is limited by its single-center design, relatively small sample size, and lack of angiographic correlation, which may limit generalizability and detailed mechanistic interpretation of thrombolysis outcomes.

## Conclusions

This study demonstrates that young adults constitute a significant proportion of STEMI cases and exhibit a distinct risk factor profile characterized by a higher prevalence of smoking and lower prevalence of traditional metabolic comorbidities. Despite comparable clinical presentation and timely thrombolytic therapy, younger patients demonstrated a significantly lower rate of successful thrombolysis, suggesting potential age-related differences in thrombus characteristics and reperfusion response. No statistically significant difference in short-term clinical outcomes was observed between the two age groups; however, the study was not powered to detect clinically meaningful differences in ACVE or mortality. These findings highlight the importance of targeted prevention in younger populations and suggest that further studies should evaluate whether pharmacoinvasive or early invasive approaches improve reperfusion outcomes in young STEMI patients.
